# Implantation of a 3D-printed titanium sternum in a patient with a sternal tumor

**DOI:** 10.1186/s12957-018-1315-8

**Published:** 2018-01-15

**Authors:** Anton Dzian, Jozef Živčák, Rastislav Penciak, Radovan Hudák

**Affiliations:** 1 Thoracic Surgery Clinic, Jessenius Faculty of Medicine in Martin, Comenius University in Bratislava, Kollárova 2, 036 59 Martin, Slovakia; 2 Department of Biomedical Engineering and Measurement, Technical University of Kosice, Letna 9, 04200 Kosice, Slovakia

**Keywords:** Sternal tumor, Sternectomy, 3D–printed titanium sternum

## Abstract

**Background:**

Primary malignant or metastatic sternal tumors are uncommon. A subtotal or total sternectomy can offer a radical form of treatment. The issue is to restore the structural integrity of the chest wall.

**Case presentation:**

We report the implantation of an individualized 3D–printed titanium sternum in a patient with a sternal tumor.

**Conclusions:**

We believe that tridimensional print technologies may also change the strategy of chest wall reconstruction.

## Background

Primary malignant or metastatic sternal tumors are uncommon. A subtotal or total sternectomy can offer a radical form of treatment. If the sternal tumor has not invaded the mediastinal structure, the sternectomy does not present a surgical technical problem. The issue is to restore the structural integrity of the chest wall. Reports on implantation of autogenous, allogeneic, and prosthetic grafts have been published. Current modern 3D printing technologies bring a change in the strategy of chest wall reconstruction. We report the implantation of an individualized 3D–printed titanium sternum in a patient with a sternal tumor.

## Case presentation

**Fig. 1:**
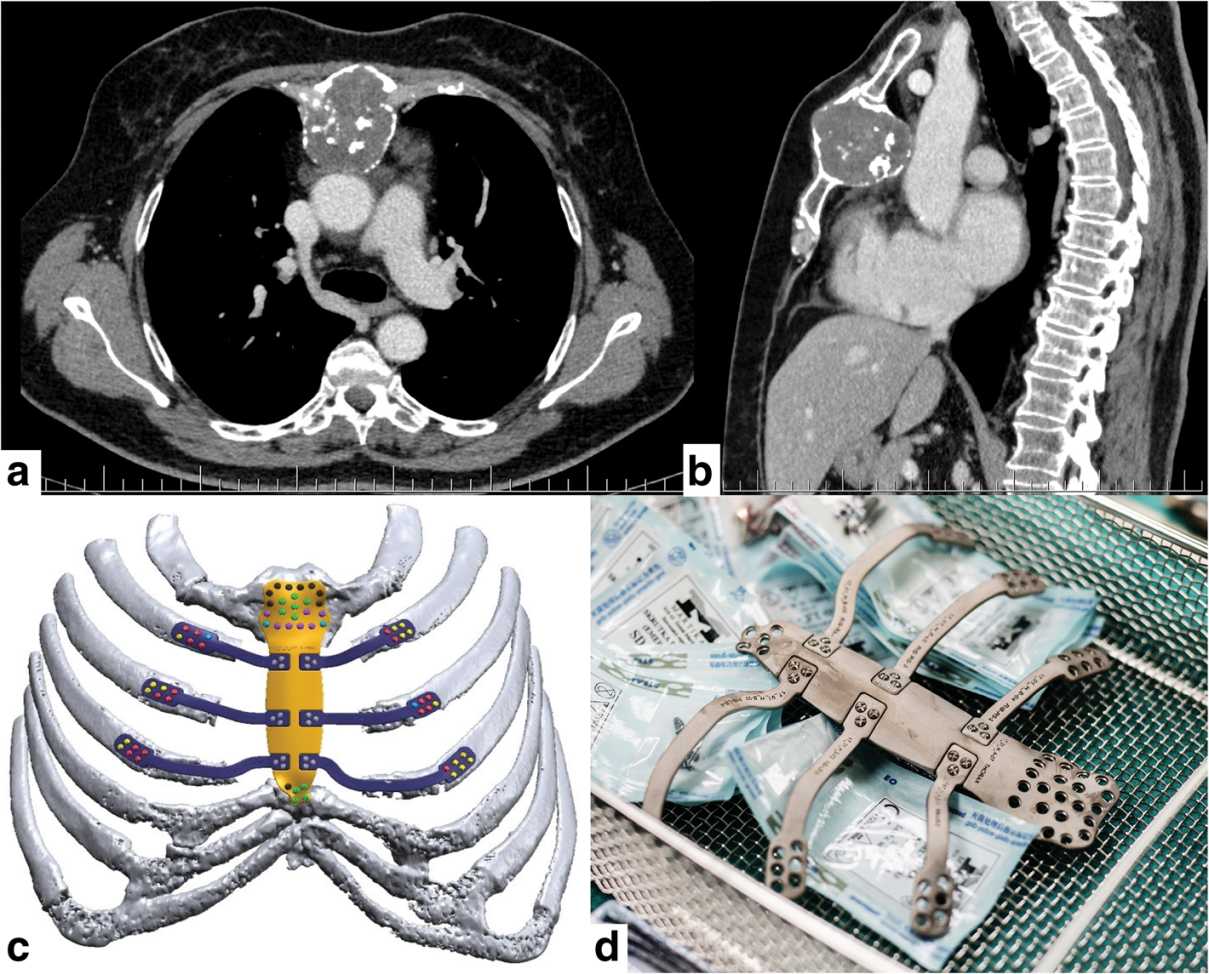
**a** Axial chest computed tomography of the sternal tumor. **b** Sagittal computed tomographic reconstruction of the sternal tumor. **c** Digital model of the implant. **d** Titanium implant ready for insertion

**Fig. 2:**
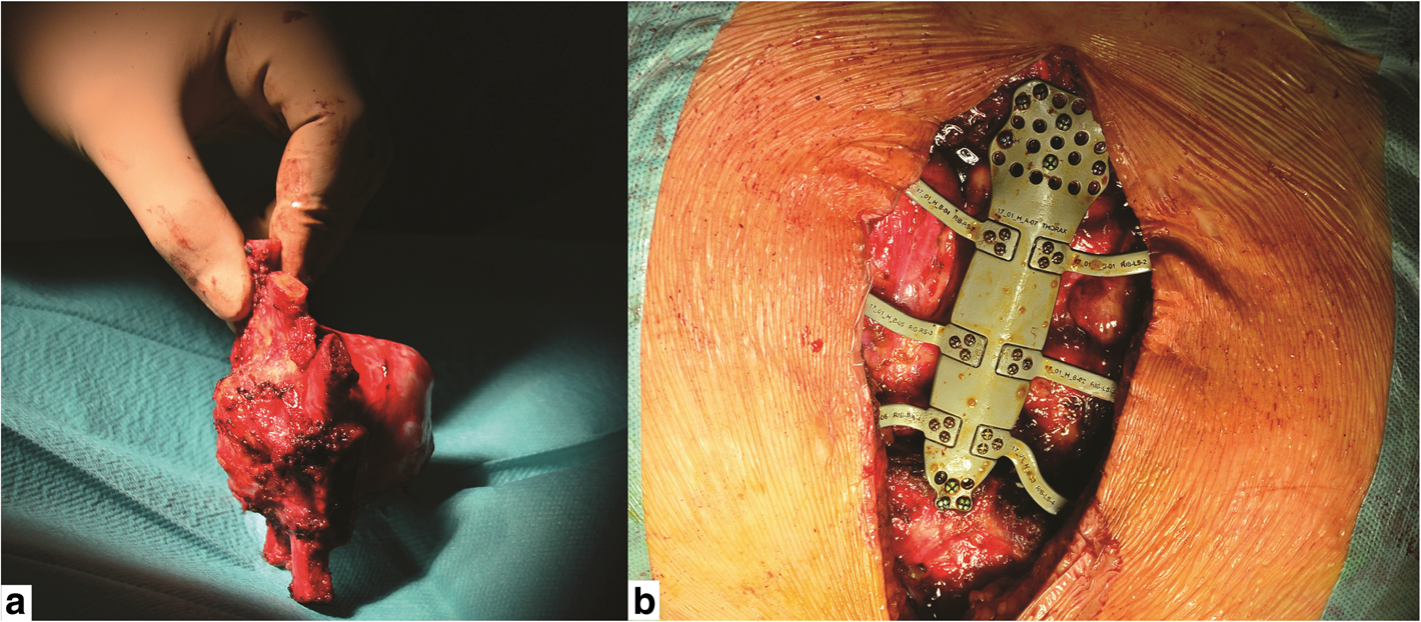
**a** Resected sternal tumor. **b** Intraoperative photograph of the implanted titanium-printed prosthesis

A 70-year-old woman with a history of colorectal and thyroid cancer, after radical operations (colon resection and thyroidectomy), chemotherapy and radioactive iodine therapy was admitted to our clinic with a sternal tumor appearing on computed tomography (CT) (Fig. 1a, b). A physical examination showed the solid mass of the sternum body. A positron emission tomography (PET-CT) scan revealed the 60 × 50 × 53 mm mass of the sternal corpus, with fluorodeoxyglucose (FDG) uptake and no other sign of local or distant metastasis. The surrounding tissue and mediastinal organs were non-infiltrated by the tumor. The best treatment option was subtotal sternectomy—resection of the sternal body with the adjacent sternocostal cartilage. Sternal reconstruction was discussed with biomedical engineers, who used source CT data and special CAD software Mimics (Materialize, Belgium) and Geomagic Design X (3D Systems, USA) to prepare a digital model of an assembly in 2 weeks (Fig. 1c), which consisted of a sternal implant and implants for the ribs. Afterwards, the implant (Fig. 1d) was additively manufactured using DMLS (Direct Metal Laser Sintering) technology (EOSINT M280 machine, EOS GmbH Electro Optical Systems, Germany) with a weight of 53.5 g and a size of 170 × 60 × 105 mm.The patient received a surgical antibiotic prophylaxis. A midline incision was made and the pectoralis major muscles were pushed laterally. We performed en bloc resection of the sternum body with approximately 3-cm pieces of cartilage from the 2nd to 4th ribs, bilaterally (Fig. 2a). The lines of the sternal body resection were 2 cm away from the tumor. Frozen section analysis confirmed the tumor-free sternal margins. The sternal implant was placed and fixed to the manubrium, the distal sternal part and bone parts of the second to fourth ribs bilaterally at additional fixation points (Fig. 2b). To join single titanium components of thoracic implant, the 18 metric screws M2.0 × 4.0 mm were used. For fixation of thoracic implant components to bone tissue of the patient; to the manubrium, the sternum, and the ribs, the SD2.8-L splint screws (MARTIKAN, Slovakia) with defined length for each fixation point were used.

For insertion of SD2.5-L splint screws, the D121 screwdriver (MARTIKAN, Slovakia) was used. The medial margins of the pectoralis major muscles were sutured together to cover the implant. A redon tube was inserted into the mediastinum and was then removed 6 days after the operation. The length of surgery was 150 min and blood loss was 200 ml. Postoperatively, the patient had no chest paradoxical motion, experienced minimal pain, and had no limitation and no regional effusion. A routine postoperative chest x-ray demonstrated no pathology. The patient was discharged on the 10th postoperative day. Histopathology revealed low-grade chondrosarcoma.

## Discussion

The principle of sternal resections and reconstructions is an adequate radical resection associated with the maintenance of chest stability for lung function as well as an acceptable cosmetic result [1]. A critical point in anterior chest wall reconstruction is a suitable prosthetic replacement that is able to restore the rigidity of the chest and prevent paradoxical motion [1]. Today, no consensus exists on which is the ideal prosthesis material and the optimal technique to reconstruct chest wall defect, and the decision still remains on the surgeon’s choice [1].

Synthetic materials such as polypropylene mesh, polyester mesh, polytetrafluoroethylene mesh, expanded polytetrafluoroethylene mesh, polyglactin 910 mesh can be used to reconstruct the chest wall defect after resection. They are relatively cheap and have high affinity for tissue growth, but its lack of rigidity in patients with extensive defects may result in paradoxical motion of the chest wall [2].

Methylmethacrylate is an interesting prosthesis material. It can be used alone or incorporated between two layers of polypropylene mesh in a sandwich fashion. Methylmethacrylate is lightweight, versatile, with minimal cost, and penetrable by x-rays. Loosening with dislocation and fracture of a chest wall sandwiched methyl methacrylate prosthesis were reported [3].

The biomimetics used with CAD modeling are pursued by respecting the anatomy, preserving function, selecting adequate reconstructive materials and integrating multidisciplinary efforts for complex reconstructions [4]. An individualized approach is necessary in modern chest wall reconstructive surgery. Technological advances in tridimensional printing using the principles of biomimetics enable production of 3D–printed titanium sternum. Recently, three modern case studies have been published on the application of a custom made titanium implant after anterior chest wall resection [5–7].

The titanium alloy and 3D technology used have practically unlimited possibilities in terms of the desired shape and size of the individualized implant, based on the specific patient anatomy. The prosthesis material is characterized by high strength while maintaining low weight and excellent biocompatibility, which guarantees minimal potential complications. The risk of migration or dislocation is limited by optimal planning of the number and location of fixation points. Preoperative individualized modeling of the implant enables easy insertion and fixation, which reduces the operation time. The short-term results were excellent: postoperative pain was minimal; the functions and stability of the chest remained unchanged; the cosmetic effect was fine and there was no regional seroma. Our recent and published data are insufficient to assess the long-term outcomes.

## Conclusions

A 3D-printed titanium implant meets the criteria for an ideal prosthesis: user-friendliness in its placement and in adaptation by the patient’s body, without significant functional limitations and with an adequate cosmetic effect, biocompatibility, minimal risk of infection, high strength with minimal possibility of mechanical failure, low weight and ideal individualized material for fixing with preoperative planning of fixation points. The technology of individualized 3D printing is very well applicable for the reconstruction of chest wall defects. It reduces morbidity and brings benefits for the patient in terms of optimal function and structural chest configuration after resection.

## Abbreviations

CT: Computed tomography
DMLS: Direct metal laser sintering
FDG: Fluorodeoxyglucose
PET: Positron emission tomography
